# Medicine

**Published:** 1920-06

**Authors:** J. M. Fortescue Brickdale


### Progress of the Medical Sciences.

### MEDICINE.

**Treatment of Gastric and Duodenal Ulcers.**—There are now three distinct lines of treatment in cases of gastric ulcers, apart from surgical interference, each of which it is claimed produces a large proportion of cures. Friedenwald states that in his experience 72 per cent. of cases treated on the old lines of rest and very restricted diet recover. Under the Lenhartz treatment —in which a somewhat liberal diet is allowed and the patient is kept in bed only for four weeks—66 per cent. of cases recover. Of patients treated on the system elaborated by Sippey of Chicago 86 per cent. recover. Sippey considers that the main factor which prevents a gastric ulcer from healing is the presence of the hyperacid gastric juice, and his method of treatment consists of an attempt to keep the gastric hydrochloric acid neutralised by frequent feeding and the administration of large quantities of alkalies. Three ounces of a mixture of equal parts of milk and cream are given every hour from 7 a.m. to 7 p.m. After a few days soft-boiled eggs and cereals are gradually introduced, so that in ten days the patient gets an egg or three ounces of a cereal food alternately added to every other milk feed. Other soft, palatable foods are subsequently employed, provided that they are not excitants of gastric secretion. Alkaline powders, such as magnesia and sodium bicarbonate in 10 grain doses are given between the feeds ; several doses are also given at night, and if there is excessive secretion during the fasting period (7 p.m. to 7 a.m.) the stomach contents are evacuated by aspiration. Hurst, who speaks highly of this method, prefers calcium and bismuth carbonates in 10 and 30 grain doses respectively, because they act slowly and give rise to no secondary hypersecretion. He also gives half an ounce of the latter salt suspended in water two hours before the first morning feed, after which the patient lies on his right side for a time, in order to bring the salt in contact with the ulcer. He recommends evacuating the stomach contents at 11 p.m. (if more than two ounces is present), and if half a pint or more can be obtained the stomach is again emptied at 1 a.m. He also uses atropine sulphate hypodermically during the night to inhibit secretion. The treatment takes about four weeks, and after that time the alkaline powders must be continued for some months, and all substances known to excite copious gastric secretion must be avoided.

In cases where there is much vomiting Friedenwald employs Einhorn's duodenal tube. This instrument is passed into the stomach and left in position, and after an hour or two is carried by peristalsis into the duodenum. The patient is then fed every two hours through the tube with any liquid food. About three ounces are given at first, and gradually increased to nine or ten ounces at each feeding. Einhorn's tube is fitted with a special syringe, by which the food is given without disconnecting the tube. Care must be taken to keep the food at body temperature and quite liquid ; the tube remains *in situ* for ten days.

Friedenwald also recommends scarlet red in addition to alkaline treatment ; it is, he thinks, especially useful in cases where the patient is up and about and the ulcer not presumably acute.

Hurst applies Sippey's method to both duodenal and gastric ulcers, and also insists on the importance of eliminating all forms of sepsis—oral, appendicular or connected with the nasal sinuses.

George Hayem has employed kaolin in doses of 20 grains a good half hour before food as a substitute for sub-nitrate of bismuth. As the powder is lighter than bismuth it is important to give it time to settle on the mucus surface before food is introduced. If well washed it is practically tasteless, but if preferred may be flavoured with a little anise or peppermint. Hayem does not consider kaolin superior to bismuth, which in the form of subnitrate prepared according to the old French codex he believes a most valuable drug. But he employs kaolin owing to the high price of bismuth salts, and owing to the difficulty of obtaining a subnitrate which is not too acid.

**Surgical Treatment of Gastric Ulcer.**—Friedenwald and Finney have observed better results after pyloroplasty and pylorectomy than after gastro-enterostomy, both immediate and final. The latter procedure, on account of its unsatisfactory end-results, they would employ only for malignant disease with pyloric stenosis. Hurst would limit operative interference to (1) perforated ulcer; (2) pyloric obstruction, (*a*) without symptoms of active ulceration, (*b*) with symptoms of active ulceration, if there is still well-marked stasis after ten days of Sippey's treatment; (3) organic hour-glass constriction; (4) severe recurrent cases which resist medical treatment; (5) severe recurrent hemorrhage; (6) if there is the possibility that the ulcer is becoming malignant.

**The Chronic Dyspepsias of the "Gassed."**—French writers during the war laid more stress on the immediate effects of poison gas on the alimentary tract than did either English or German observers. Achard noted as late effects hyperchlorhydria, chronic diarrhœa, and other digestive disturbances in a certain number of cases. The report of the Chemical Warfare Medical Committee, however, found that in cases of persistent vomiting there was hypochlorhydria with normal motility, and that the condition was undoubtedly a neurosis. Lœper found late digestive disturbances not infrequent, and most usually due to chlorine or mustard-gas, and not so often to purely lachrymatory gases (xylyl bromide, etc.). He divides the cases into two types, (1) the flatulent type, with loss of appetite, distension, ærophagy and discomfort an early syndrome; (2) the painful type, in which the symptoms occur later, and are accompanied at times with very severe pain. Intermediate cases also occur. Both hyper- and hypochlorhydria occur, the former usually being associated with a desquamative gastritis and the latter with the exudation of large numbers of lymphocytes both polynuclear and mononuclear into the stomach. But apparently this relationship is by no means invariable. Lœper considers these findings conclusive evidence of an organic lesion, and for the most part dismisses the obviously neurotic manifestations as secondary. He has seen no case of true ulceration, but in three instances found blood in the stomach contents by microscopical examination. In three cases he discovered persistent deformity of the greater curvature, and in one partial stenosis of the pylorus. Lœper had no opportunity of examining the stomach in any of his cases either at an operation or autopsy, and found his view of the organic nature of the disturbance on the microscopical examinations of the gastric contents. His first type shows large quantities of desquamated epithelial cells in the gastric contents, his second type numerous leucocytes; these he correlates with a superficial and deep inflammatory condition respectively.

### Reviews of Books.

